# Lost in Translation: Transforming Behaviour Change Techniques into Engaging Digital Content and Design for the StopApp

**DOI:** 10.3390/healthcare6030075

**Published:** 2018-07-06

**Authors:** Emily Anne Fulton, Kayleigh L. Kwah, Sue Wild, Katherine E. Brown

**Affiliations:** 1 Centre for Advances in Behavioural Science (CABS), Coventry University, Coventry CV1 5FB, UK; kayleigh.kwah@coventry.ac.uk (K.L.K.); Katherine.brown@coventry.ac.uk (K.E.B.); 2 Public Health Warwickshire, Warwickshire County Council, Warwick CV34 4RL, UK; suewild@warwickshire.gov.uk

**Keywords:** digital, health behaviour change, intervention, engagement, design, behaviour change techniques

## Abstract

Frameworks to support the application of behaviour change theory to the choice of behaviour change techniques (BCTs) in designing digital behaviour change interventions (DBCIs) are becoming well established, and have been employed by the authors in the development of StopApp. However, guidance on the next stage—effective operationalisation (translation) of these BCTs to a digital context, including the precise delivery and design of “behavioural intervention technology” (BIT) elements, is still in its infancy. This is despite growing recognition of the need to optimise engagement and usability, alongside a theoretical basis, for intervention effectiveness. The aim of this study was to explore methods to translate BCTs into digital content in an accurate and systematic manner. We describe the process of using co-creation (user-led) rather than expert-driven methods in the development of user-facing features and design in StopApp, including the iterative “bottom-up” and “top-down processes” necessary for accurate BCT translation. We found a small disparity between the intended and actual BCT content, reflecting the difficulties of translating BCTs into digital intervention content and the need for better guidance and methodical approaches to enhance this under-researched process. The involvement of our Patient and Public Involvement (PPI) group throughout these processes is described.

## 1. Introduction

It is well recognised that despite the ongoing proliferation of digital behaviour change interventions (DBCIs), relatively few have a clear theoretical basis, designed and developed methodically to optimise user uptake, engagement, and sustained behaviour change. Two issues present themselves. Firstly, although DBCIs benefit from an increasingly accessible and scalable mode of delivery, they also incur the challenge of applying and translating theory commonly intended for non-digital application, into something that performs in a digital format. Secondly, users of health apps typically decide whether to engage with them in a mere 50–500 milliseconds [1], regularly ceasing use shortly after download [2]. Identifying what leads potential users to develop interest with a DBCI (uptake), and what enhances usability so they “stay with it” (engage) enough to activate the target behaviour change, is imperative. Understanding the minimal engagement with a DBCI (level and pattern of usage) necessary to establish the desired behaviour change outcome is essential, in order to maximise success [3]. In order to achieve this, evaluations of DBCIs should collect real-time objective usage data to fully understand the patterns and mediators of use [4,5]. Engagement with technology has been defined as the “quality of user experience characterized by attributes of challenge, positive affect, endurability, aesthetic and sensory appeal, attention, feedback, variety/novelty, interactivity, and perceived user control” [6]. The “technology acceptance model” [7] argues that attitudes towards, and behaviour (interaction) with a technology are influenced by its perceived usefulness and ease of use. Alongside cognitive predictors of engagement, the importance of emotion in the adoption and continued use of digital interventions has been highlighted [8].

### 1.1. Optimising Engagement for Behaviour Change

The literature suggests that a number of key design principles can be applied to DBCIs to optimise uptake and engagement for improved outcomes. Eleven engagement features and nine ease-of-use features have been identified [9], including content to establish rapport and clear expectations with users early on, transparency about what the DBCI is trying and not trying to do; and design parameters, such as reducing the need for scrolling, highlighting key terms and avoiding excessive and small text. DBCIs are encouraged to avoid childish or amateurish design [10]; use chatty everyday language that avoids a patronising tone [11]; offer tailoring within the intervention [12]; provide a sequential and intuitive user experience requiring minimal effort; and use icons rather than text where feasible [13]. A series of “gambits” (design patterns) have been proposed for improved design, thought to influence user behaviour, including the use of memes (images or text that are shared via social media to increase reach and uptake), and the use of “Kairos”—the provision of suggestions to act at the opportune moment to change behaviour [14]. For example, when a user first logs on to a digital intervention, a pop-up box opens at this well-timed moment inviting them to take a virtual tour.

**Figure 1:**
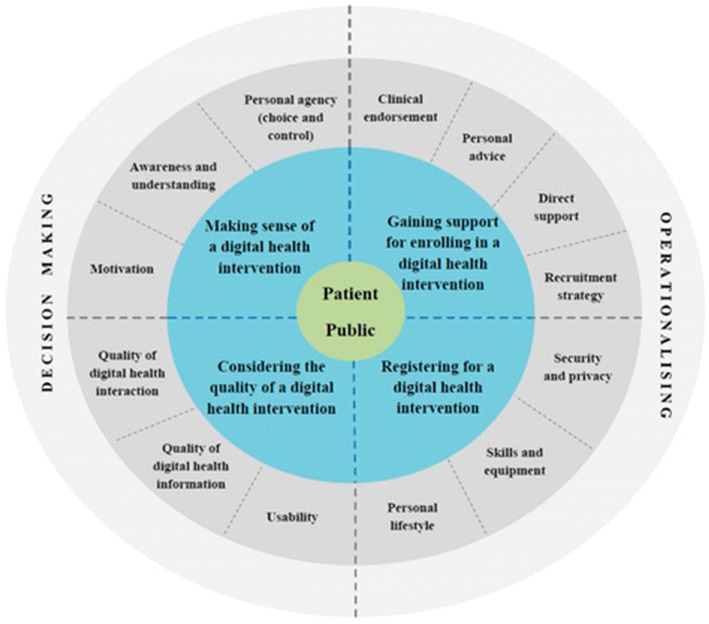
Digital health engagement model (DIEGO).

In a systematic review of 19 studies, key factors contributing to successful engagement and recruitment to digital health interventions (DHIs) were identified [15]. These included “engagement strategies” such as indirect advertising (e.g., online, electronic, and print media) and direct personal contact; and “recruitment strategies” such as automatic, electronic, paper-based, assisted (health professional creates an account) or via the phone. The findings were used to develop the “Digital health engagement model (DIEGO)” (see Figure 1), which outlines four processes for improving user engagement: (1) “Making sense of the DHI”, including understanding the recruitment message, awareness about the benefits; and perceived control and choice in use; (2) “Considering the quality of the DHI”, including the perceived quality of the intervention, trust (openness and honesty), the quality of information given, and the level of complexity for use (whether interactions were automated and integrated); (3) “Gaining support for enrolling in the DHI”, including recommendations from trusted sources and clinical endorsement; and (4) “Registering for a DHI”, including factors such as digital literacy, security concerns, and internet access.

### 1.2. A Digital Behaviour Change Intervention: StopApp

StopApp is a digital web-app intervention designed to enable greater access and attendance at the UK National Health Service (NHS) Stop Smoking Service (SSS). Attending a service increases a smoker’s chances of quitting fourfold [16], however, rates of attendance in recent years have been in decline [17]. Booking an appointment requires individuals to know where and how to access services, placing unnecessary barriers in the way of access. StopApp is accessible via all digital platforms and does not need to be downloaded, but looks and functions like an app on a Smartphone. In its most simple form, the aims and content of StopApp can be illustrated using the “MAP” (defined below) acronym to describe three routes to behaviour change within the “Health Behaviour Change Competency Framework” [18]. These include (i) **M**otivation; (ii) Enabling **A**ction and (iii) **P**rompting behaviour without having to engage in thinking. StopApp includes a brief evidence-based behaviour change component designed to enhance motivation to attend and re-frame negative beliefs or a lack of awareness about Stop Smoking Services. Content includes a series of “behaviour change techniques” (BCTs), the smallest active components of an intervention that are capable of changing behaviour (see [19] for the full taxonomy).

StopApp aims to encourage the target behaviours (booking and attending at Stop Smoking Services) without the need to process a lot of information and engage in lengthy decision-making processes. The series of BCTs are delivered alongside a quick and easy booking system, whereby the individual can choose a location, date, and time; and instantly book an appointment, receiving confirmation texts and reminders to attend. The aim is to prompt “behaviour without thinking”, especially important as research suggests smokers unmotivated to quit are most likely to respond to low effort and short communications in attempts to initiate quitting [20].

Unlike most health apps and interventions, StopApp is designed for one-off use, encouraging users to book from the first page, and every page onwards with a fixed link. Some users may open StopApp, aware of its purpose, with the intention to book immediately, others may be unsure what StopApp is, or what an appointment at services entails, hence the goal of the content is to encourage users to engage with it for long enough to increase their motivation and take action to book. Four points of behaviour change are therefore necessary: (i) accessing StopApp (uptake); (ii) interacting with StopApp (engagement); (iii) booking an appointment; and (iv) attending an appointment.

### 1.3. Translating Behaviour Change Techniques (BCTs) into Digital Content and Functionality

The literature contains relatively sparse guidance regarding the optimal parameters for the digital development and design of DBCIs, in order to incorporate behaviour change theory within the design and operationalisation of content. The choice of which behaviour change techniques (BCTs) to include in StopApp resulted from an extensive exploration of the barriers and facilitators to Stop Smoking Service uptake and extensive “behavioural analysis” using the Behaviour Change Wheel framework [21] (see [22] for a full description of this process). BCTs delivered in digital format depend on their design, dose, and duration, as well as content, to optimise effectiveness. The most well thought out and comprehensive BCT is of little use if it fails to develop interest with attractive design, or is not user-friendly and intuitive to use in terms of digital functionality. Therefore, the method of intervention content delivery—effective operationalisation of BCTs and the resulting elements and features within an intervention, is a crucial and currently under-recognised process within intervention development. Although no agreed definition exists, the term “Form of Delivery (FoD) elements” have been proposed to describe these BCT elements and features [23]. Incorporating far more than mere “mode of delivery”, these elements suggest the need to consider details about the provider, format, materials, setting, intensity, tailoring, and style, alongside the different configurations of BCTs (frequency, intensity, duration), as these are deemed of equal importance for intervention success as the prior theoretical basis and BCT choice.

### 1.4. Co-Creation Methods to Enhance Engagement with DCBIs

One way to increase engagement is in the use of co-design and co-production (“co-creation”). As opposed to an expert driven approach whereby professionals or researchers drive the design and content of an intervention, “co-creation” involves end users in the process, enabling insights about the target audience that are previously unknown, resulting in very different intervention content and design [24]. 

Aims: The aim of this study was to translate behaviour change technique (BCT) concepts into digital content within StopApp, in a systematic and accurate manner utilizing co-creation methods, and to assess the effectiveness of this process using bottom-up and top-down approaches. Bottom-up approaches refer to methods whereby there is a systematic move from individual elements to the whole, for example, ensuring intervention content is all-inclusive and nothing is missing. In relation to this study, this describes the systematic re-assessment of BCTs and subsequent content, whereby the researchers ensure that all relevant BCTs are included, more added if needed, before the final intervention is agreed. Top-down approaches refer to methods whereby the whole (e.g., a complete digital intervention) is then broken down into its parts (e.g., the BCTs) to gain insights on what has already been formulated/designed. In relation to this study, this approach was taken when the final StopApp content was recoded for BCT content by the two independent researchers. They code only what is there and add in nothing new. We describe the process by which the content of StopApp was drafted, tested and revised; a single use intervention aiming to engage a relatively hard to reach group—smokers. Continuous end-user involvement throughout the design process is described. This “person-centred approach” to digital intervention design ensures user involvement beyond assessing “acceptability, usability, and satisfaction” [25], such that end users, including staff involved in implementation and dissemination of the DBCI, are involved in and consulted on every draft of the design and content. In particular, we sought to ensure the accurate translation of the BCTs into digital elements, which required multiple assessments and coding of BCT content, to ensure fidelity to BCT descriptors, as described in the Behaviour Change Wheel (BCW) handbook [21], alongside optimal user engagement.

## 2. Methods

Ethical approval was granted from Coventry University Research Ethics Committee. To avoid replication, the processes employed for BCT operationalisation, content generation, and web development processes are described in the results section.

### Co-Creation and User-Testing

To ensure the content, usability, language and pitch was appropriate for the target user, we involved a “Patient and Public Involvement” (PPI) group in all stages of the design process, from idea inception to BCT content generation, logo design choice, functionality and written content. This included smokers and ex-smokers, and also an “expert panel” of Stop Smoking Service staff and advisors, Tobacco Control commissioners and Public Health officers. The PPI group (*n* = 7) was recruited via social media channels, community settings, and the NHS Stop Smoking Service. The group consisted of four males; and current and ex-smokers, of which one had used the Stop Smoking Service. All PPI group members were given information about the study and asked to provide written consent in order to take part. A model of “participatory design”, whereby end users act as co-creators was used [26], focusing on “person-centred approaches”—an approach focused on the needs of the individual, as paramount. This included the following stages:

(i) Involvement of the PPI group in the generation of web-app features for delivering and representing BCTs in StopApp that were identified in the previous study (see [22]). For each BCT, the study team and web developers discussed and agreed on potential “operationalisations” (the translation of BCT criteria into measurable digital functions or content). Ideas for how this would be represented specifically in StopApp (e.g., exact text, functions, design, dose) were generated, and numerous possibilities discussed with the study team, expert panel, and PPI group. These were termed “behavioural internet technology” (BIT) elements, as described in the “Behavioural Intervention Technology Framework” [27]. The list was then subject to a further assessment using the “APPEASE” framework to guide decision making (e.g., consideration was given to “affordability, practicability”, “effectiveness/cost-effectiveness”, “acceptability”, “side-effects/safety”, and “equality”) [28].
(ii) Creating and testing the prototype—Input from the PPI group was given on three revisions of StopApp using early paper draft content, wireframes, and a final digital draft prototype. Workshops were used to explore thoughts regarding (i) functionality; (ii) navigation and usability; (iii) engagement; (iv) acceptability; (v) content. This included a “think aloud” session with members of the PPI group. Five participants (4 male) took part in these one-to-one, face-to-face sessions. Two males were ex-smokers, and the rest were current smokers. Although age was not recorded, they ranged in age from early twenties to late forties. The lead researcher explained the purpose of the study and procedure for the session, followed by participants being asked to view and move through the pages of the web-app themselves, with no direction, talking out loud about their thoughts regarding where to click, the functionality, ease of use, content, and appearance. A second researcher made notes regarding body language and any difficulties participants appeared to encounter in terms of use, for example, technical glitches and uncertainties about how to move forward, or where to click. The sessions lasted 30–45 min and were audiotaped.
(iii) PPI feedback was given regarding the logo, fonts, colours and branding (stylesheets) to be used for StopApp.

## 3. Results

At the final stage, a late prototype of the StopApp was subject to a re-coding of BCT content by two independent researchers who were blind to the intended BCT content, and had not been involved in the StopApp development process. This was to ensure that the translation of BCT theory was operationalised accurately following the many revisions that took place, by comparing the end digital (app) content with the original theoretical intended (BCT) content.

### 3.1. Exploration and Identification of Behaviour Change Technique (BCT) Representation in Digital Format & Assessment of Suitability for a Digital Format

BCTs identified in the previous intervention content development study [22] were reassessed and discussed with the study team, alongside all 93 BCTs within the BCTTv1, to consider whether any potential content or functionality had been overlooked. It was agreed that no further BCTs could or should be applied, especially given that this “top-down” approach meant their inclusion was not based on the results of the behavioural analysis (evidence and theory) (see [22] for a description of this process). For each BCT, a number of ideas about how it could be translated into app features and content, and how these could be delivered digitally, were generated. This required continuous reference to the BCT criteria outlined in the BCTTv1, and discussions about whether draft content still met the BCT criteria in a digital context. Alongside input from the PPI group, three BCT’s (10.5 Social Incentive, 5.5 Anticipated regret and 15.3 Focus on past success) were excluded at this juncture as the relevant content was deemed unsuitable and patronising. Social incentive was removed because it was identified as illogical that users would be told in advance they would receive a congratulatory message on booking and attending. Anticipated regret was removed because the wording was considered to be patronising and Focus on past success was removed because the wording no longer fitted the BCT criteria. Another BCT (11.2 Reduce Negative Emotions) was added when it was recognised that content developed now met this criterion (for example, quotes from ex-smokers who had used Stop Smoking Services demonstrated how fears of attending disappeared when they met the advisor). The mode of delivery for some BCTs was also revised, for example, videos of smokers who had used services were not included, due to issues with affordability, practicability, and cost-effectiveness; having recognised that this would be expensive to create and stream, and impractical to watch on the move. The DIEGO model was used to guide similar decisions regarding the user journey. For example, security and privacy issues were discussed, along with the need to include clinical endorsement from the NHS without discouraging users. Ideas about how the BIT elements would be represented in the app including content, functions, and design were listed and revised several times following PPI feedback. Examples of BCT operationalisation and related BIT elements can be found in Table 1.

### 3.2. Visual Design and Front-End Development

Once the list of relevant behavioural intervention technology (BIT) elements were identified, a paper storyboard was created between the study team and web developers, to outline how the BIT elements would be represented in a sequential journey for the end user. It was agreed at this stage that the booking facility would be clearly prominent as a link at the bottom of every page to encourage booking. The order of BCT presentation was redrafted several times with further input from the PPI members. Based on the need to present minimal text relevant to the individual, content was reduced, and icons created to deliver messages, tailored based on what users identified were reasons or concerns about attending Stop Smoking Services. The content and functionality were then developed into a series of digital “wireframes” by the web developers, and subject to further user testing by the study group, PPI group, and expert panel.

### 3.3. Think Aloud Sessions

Overall, participants commented that they felt the content was good, but not delivered in an appealing way, with a relatively poor structure and too much text, although the navigation was deemed straightforward, requiring little direction. Participants liked the testimonials, facility to have StopApp sent as a reminder later on, the provision of appointment time, and date choice, and encouraging messages without excessive pressure to book. Participants felt that StopAppv1 needed more guidance, at the onset, about what the aims were and what it was trying to do. The message that smokers are four times more likely to stop with help from services was deemed important, and should be presented in a more bold and striking way for impact (e.g. an infographic). One participant felt design improvements were needed: “it looks like a PowerPoint presentation”. Others felt there was too much to read, too much white space, and too many clicks required, and the logo should be present on each page but made far smaller as it dominated at present. The need for a trustworthy known logo (e.g., NHS/University) was also suggested. Participants felt the buttons were unclear and required further explanation, that the “back” and “home” buttons were in the wrong place, and the use of a triangle arrow continue button was misleading, as it looked like a “play” button. Participants suggested that the booking feature sent an automatic appointment to the user’s outlook or other phone calendar. Within the booking facility, they suggested that locations for services should be listed by distance from the input postcode or location, as is common practice in similar online tools. They also suggested that the map provide the service names rather than numbers, to improve speed of use. One participant felt it was unclear how to go back to search for a new service, therefore, available options should be made more visible and easy to access. In terms of design and appearance, it was suggested that the graphics used were too comical and made “a serious message look like a joke”.

Web design principles to optimise engagement and, most importantly, behaviour change, were also explored with the PPI group, expert panel, and study group, considering, at every step of the user experience, what would make users more likely to click “book”, and what might detract them. Examples of design considerations from the literature, study team discussions and the PPI group, are illustrated in Table 2.

### 3.4. Back-End Development & Testing the Interoperability between StopApp and the Stop Smoking Service (SSS) Electronic System

**Figure 2:**
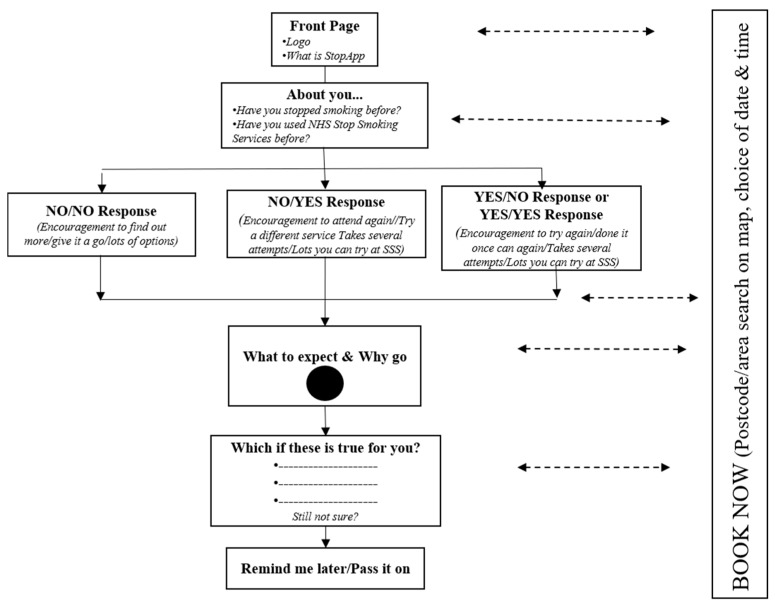
Flowchart of basic StopApp structure.

In order for StopApp to enable instant booking at services, it was necessary to integrate StopApp with the existing service booking systems used locally in Warwickshire (and increasingly nationally). This is offered by two systems, for pharmacies “Pharmoutcomes”, and GPs “Outcomes4Health”, created by Pinnacle Health Ltd. To create a seamless user experience, users choose an available appointment within StopApp, and in order to book this, their name and mobile number are exported into Pinnacle’s system to register the booking on an online calendar visible to staff in the services. This enables staff to contact the user in the unlikely event of staff illness/the need to rearrange an appointment. Data is not stored on StopApp, therefore ensuring security and information governance requirements are met. A digital calendar system did not exist within the pinnacle system used by services, therefore, StopApp commissioned Pinnacle Healthcare Ltd. to create this within their existing system. This was achieved with input from the lead commissioner for services and tobacco control within the local public health department. Feedback from several Stop Smoking Services was also sought to ensure usability for the advisors receiving the bookings. Based on this feedback, additional functions were added, and existing tools amended accordingly. For example, advisors requested that clinic times could be entered as a recurring (rule) or standalone event for ease, and that the tabs were relabeled for clarity. Back-end functionality testing was conducted to ensure the functioning of the booking system, and that confirmation and reminder to attend messages were generated. StopApp was viewed on tablets, android/iPhone, and desktop computers to test viewing compatibility across platforms. The basic structure of StopApp content and navigation is illustrated in Figure 2.

### 3.5. Top-Down Coding of App Content for BCTs

Agreement with the existing BCT list between the two independent researchers was high, however, the second coding identified two additional BCTs that the coders felt were present that the study team, expert panel and PPI group had not identified (10.7 Self-Incentive; 10.4 Social Reward). 10.7 Self-Incentive was not added following re-discussion within the study group, the independent researchers, and referral to the BCTTv1, as it was not deemed feasible to help users plan a future reward on booking; however, 10.4 Social Reward was deemed appropriate to add due to the presence of a congratulatory message after booking an appointment. Two BCTs were not identified by the second coders (15.3 Focus on Past Success; and 16.3 Vicarious Consequences), and it was recognised in discussions that these were necessary elements, however, the operationalisation in StopApp was not accurate or clear enough, therefore, content was revised to ensure it met the criteria more distinctly. For example, a quote about quitting smoking in the past that was attributed to “15.3 Focus on Past Success”, was in fact not relevant to the target behaviour of StopApp (booking and attending Stop Smoking Service), therefore, an alternative quote about the achievement of having been to a service before and how individuals could do this again, employing an alternative method to quit, was instead included. This also highlighted the need to be very clear in the process of BCT operationalisation about what the target behaviour is. All too often it was easy to forget that the target behaviour for users of StopApp is booking and attendance at Stop Smoking Services, and not stopping smoking.

## 4. Discussion

This study aimed to effectively translate theoretical content about health behaviour change theory (BCTs) into digital app content in StopApp (“behavioural internet technology (BIT) elements”), in a systematic manner. Two additional BCTs were added in the design and development process, one by the PPI group, and the other following the second coding process. This co-creation approach therefore added content to the existing research and theory (expert-driven) approach developed in the previous study, suggesting the value of considering user person-centred approaches to enhance intervention development. This bottom-up approach to the translation of BCTs into digital content, enabled new ideas to be considered and added, rather than involving end users simply in the end product evaluation. This study also found a discrepancy between the re-coded BCT content of the final version of StopApp, and our intended BCT content at the start of the study, which was, in large part, due to the translation of content into digital design. This “top-down” approach ensures any discrepancies are checked by blind re-coding of BCT content, and amended after DBCI creation to improve the quality of BCT translation and fidelity to the BCT content and parameters outlined in the BCTTv1. Person-centred design, combined with iterative and continuous user testing (co-creation approaches) revealed many important and necessary changes that were needed and would otherwise have been overlooked. Consequently, input from a PPI group at all stages of content generation and design should be ubiquitous in DBCI development.

### 4.1. Limitations

The study is limited by the small sample size, younger age, and predominantly male participants in the PPI group. The process of translating BCTs into content is open to interpretation and bias, in particular, when weighting the influence of the literature, PPI group, and expert panel regarding what to include and exclude, and how digital content is displayed. The goal of a top-down approach after initial content was an attempt to overcome this issue. Future systematic testing and evaluation of these methods is needed in order to improve the reliability of translation and final intervention content.

### 4.2. Future Directions

StopApp will shortly undergo further investigations to test the feasibility of recruiting smokers and the processes required for a full research trial. Although recruitment to a research trial differs to real world use, iterative user evaluation will be used to explore the best messages and mode of delivery for optimal uptake and engagement with StopApp. The feasibility trial will include a larger scale user evaluation of the content, look, and functionality of StopApp. Using real-time analytics data, it will provide an assessment of BCT exposure and level of engagement, collecting data on usage patterns and behaviour at an individual level. This will enable an understanding of which pages or extent of exposure to content led to the most bookings, and at which point, users are most likely to cease use without booking. The feasibility trial will also assess undetected effective engagement—how many people engage in the target behaviours (book and attend at services) as a result of exposure to StopApp, but do not book via the app, instead using alternative means. We will measure this by requesting that all Stop Smoking Service Advisors ask what prompted service users to book an appointment, and how they heard about the service (with options including word-of-mouth, information in a leaflet, NHS website, local website, and StopApp etc.).

## 5. Conclusions

Disparities may exist between intended BCT content at the intervention development stage, and actual measurable content following digital design and creation. This reflects the general difficulties of translating theoretical phenomena into digital intervention content, and highlights the need for better guidance and methodical approaches to enhance this under-researched process.

## Figures and Tables

**Table 1:** Behaviour change technique (BCT) representation in digital format for StopApp.

No.	BCT Code, Name & BCTTv1 Descriptor	BCT Operationalisation	Examples of Behavioural Intervention Technology (BIT) components in StopApp
1	3.1 Social support (unspecified) *Advise on, arrange or provide social support or non-contingent praise or reward for performance of the behaviour*	Provide praise for booking an appointment from a stop smoking advisors Advise on the social support they will receive from stop smoking advisors on attending	(Quote) “*I was given great support and encouragement from the stop smoking advisor*”
2	4.1 Instruction on how to perform the behaviour *Advise or agree on how to perform the behaviour* (*includes “skills training”*)	Present information on how to book (and attend) an appointment	(Text in StopApp) User given guidance to search locations on a map and prompted to choose time of choice. Name and mobile phone entered, and user clicks to confirm. Confirmation text or email sent.
3	5.2 Salience of Consequences *Use methods specifically designed to emphasise the consequences of performing the behaviour with the aim of making them more memorable*	Use design principles to create an infographic that draws attention to the message that attendance at Stop Smoking Services increases chances of stopping smoking	(Infographic in StopApp) Users see eye-catching infographic to represent the message that you are four times more likely to stop smoking with help from NHS Stop Smoking Services.
4	5.3 Information about social/environmental consequences *Provide information* (*e.g., written, verbal, visual*) *about social and environmental consequences of performing the behaviour*	Draw attention to the positive behavioural consequences of attending SSS	(Text in StopApp) You are four times more likely to stop smoking with help from a Stop Smoking Service. (Quote) “*The Stop Smoking Services have turned my life around, without the support I received I would never have succeeded*” (Quote) “*I never thought I would be able to quit, I had tried before and always failed. But this time around, thanks to Kelly my stop smoking advisor I finally stuck to it.*”
5	5.6 Information about emotional consequences *Provide information* (*e.g., written, verbal, visual*) *about emotional consequences of performing the behaviour*	Highlight the positive effect associated with the sense of achievement of booking and attending SSS.	(Quote) “*I am so much happier for doing it* (*going to a Stop Smoking Service*), *I have made myself and my family so proud*”.
6	6.2 Social comparison *Draw attention to others’ performance to allow comparison with the person’s own performance*	Prompt comparison with others who used SSS and are now non-smokers. Present short stories/quotes from ex-smokers who have used SSS to stop. Include age/gender range to aid comparison.	(Quote) “*Because it has worked for me, I believe anyone who has help will stop.*”
7	6.3 Information about other’s approval *Provide information about what other people think about the behaviour. The information clarifies whether others will like, approve or disapprove of what the person is doing or will do*	Provide quotes from Stop Smoking Advisors and GPs to show they approve of going to SSS as a positive step towards stopping smoking	(Quote from stop smoking advisor) “*I am always pleased when people come to see me to explore ways to help them quit for good. Going to a stop smoking service is an achievement in itself and the first step towards success. It’s something to be proud of!*” (Text in StopApp) 9 out of 10 smokers who’ve used a local Stop Smoking Service say they would recommend it
8	7.1 Prompts/Cues *Introduce or define environmental or social stimulus with the purpose of prompting or cueing the behaviour. The prompt or cue would normally occur at the time or place of performance*	Send users text/email reminders to attend	(Example SMS content) 24 h before appointment: This is a reminder that you have an appointment on (date) at (time) with our Stop Smoking Advisor at (location). We are looking forward to meeting you. If you need to rearrange, please call (Tel. No.) Remind me in; one week, two weeks, one month
9	9.1 Credible source *Present verbal or visual communication from a credible source in favour of or against the behaviour*	Include quote or story from the stop smoking advisor/GP about the benefits of attending at SSS.	(GP Quote) “*I am always so pleased to hear when someone has been as I know they are so much more likely to quit for good with this help.*”
10	9.3 Comparative imagining of future outcomes *Prompt or advise the imagining and comparing of future outcomes of changed versus unchanged behaviour*	Include text questions to encourage users to imagine their life as a non-smoker in the future, which is more likely if they go to SSS.	(Text in StopApp) Do you want to be a smoker this time next month? Next year? Book now and the team can help you to become Smoke free. Just picture it
11	10.1 Material incentive *Inform that money, vouchers or other valued objects will be delivered if and only if there has been effort and/or progress in performing the behaviour*	If the user attends SSS, they get free or reduced cost Nicotine Replacement Therapy (NRT). List free or reduced cost NRT as a benefit of attending	(Text in StopApp) What do the team offer …Free or cheaper stop smoking medicines
12	10.4 Social reward *Arrange verbal or non-verbal reward if and only if there has been effort and/or progress in performing the behaviour*	Praise user if they book appointment at SSS via the Digital Behaviour Change Interventions (DBCI)	(Text in StopApp) That’s brilliant, we look forward to seeing you! You are now one step closer to becoming smoke free
13	11.2 Reduced negative emotions *Advise on ways of reducing negative emotions to facilitate performance of the behaviour*	Address possible negative emotions and fear associated with perceptions about what happens at SSS	(Test in StopApp) By finding out what to expect from NHS Stop Smoking services and hearing from real people about the help and support they received we hope you will feel positive about what’s on offer and consider booking an appointment today What do the team offer … A friendly, non-judgemental welcome (no nagging)
14	13.2 Framing/re-framing *Suggest the deliberate adoption of a perspective or new perspective on behaviour* (*e.g., its purpose*) *in order to change cognitions or emotions about performing the behaviour*	Encourage user to consider that their beliefs about stopping smoking/attending SSS may be viewed in a different way	(Text in StopApp) You don’t have to stop straight away, in fact the sessions usually starts a couple of weeks before you stop. Its more about talking though your options and seeing if the support is right for you If you have never tried to stop smoking before it may not be as difficult as you think. Worried about the road ahead? It is very common for people to make several attempts to stop smoking before they stop for good. Stopping smoking, even for a short period of time, is still an achievement. With support from the Stop Smoking Service you can learn what you could do differently next time to help you stop smoking for good.
15	13.5 Identity associated with change behaviour *Advise the person to construct a new self-identity as someone who “used to engage with the unwanted behaviour”*	Include text about the benefits of being someone doing what they can to be a non-smoker by going to SSS.	(Text in StopApp) Do you want to be a smoker this time next month? Next year? Book now and the team can help you to become Smoke free. Just picture it
16	15.1 Verbal persuasion about capability *Tell the person that they can successfully perform the wanted behaviour, arguing against self-doubts and asserting that they can and will succeed*	Statements to encourage users to feel they can go to SSS because it is easy to book, convenient (choice of time/location) and they will be reminded and shown how to go. No pressure to stop smoking, if they attend they are not a failure for just going.	(Text in StopApp) Not sure where Stop Smoking Services are based? Not sure how to arrange an appointment? StopApp makes this easy. You choose the location, time and date to suit you. You don’t even have to speak to anyone. We’ll even send you a reminder.
17	16.3 Vicarious consequences *Prompt observation of the consequences* (*including rewards and punishments*) *for others when they perform the behaviour*	Highlight the benefit for family and friends if you go to SSS. Doing it for their peace of mind and health too.	(Quote) *“I am so much happier for doing, I have made myself and my family so proud”.* (Quote) “*I never thought I would be able to quit, I had tried before and always failed. But this time around, thanks to Kelly my stop smoking advisor I finally stuck to it.*”

**Table 2:** Content, functionality and design factors considered necessary for StopApp.

Content	Functionality	Aesthetic/Design
Illustrate value in using StopApp on first page & unique selling point (USP) [9] Avoid creating a defensive reaction by users (e.g., avoid health scare tactics, pressure to stop smoking) * Provide option of additional information in a menu * Tailoring—based on responses to perceived barriers to going to Stop Smoking Services [12] Include testimonials & encouraging messages * Language style—informal, non-patronising, non-clinical [11] Remove all unnecessary words * Open communication, trust worthy, clear messages [15] Text suitable for all ages, literacy level, ethnic group, social background * Accreditation & endorsement from a University/NHS [15] Recommendations from trusted sources and family/friends * Remove concerns about privacy and security of personal information (e.g., not shared with third parties). Clear statement in main text and detail in “terms and conditions” and “privacy” sections [15]	Avoid administrative burden, non-complex (automated and integrated) [13] Easy navigation and booking—link on every page Include as few clicks as possible to receive information and book appointment ** Minimal scrolling [9] Intuitive interface [13] Offer to be sent link to app at a later date (Flexible and non-directive), if they are about to log-off without booking (e.g., “Kairos” [14]) Offer facility to share via social media * Avoid prohibitive costs—e.g., videos that use data to stream * Offer choice and control over reminders (e.g., not bombarded) * Ensure optimisation of CEO for easy access to StopApp when searching **	Choose a modern professional rather than clinical/academic design * Simple, one colour * Consistent formatting and layout ** Fewer text, more images/icons * Recognisable branding and simple logo * Although a web-app, make it look like an app when viewed on phones/tablets **

* Recommendation based on feedback from the PPI group; ** Recommendation based on advice from web developers.
